# Environmental and Anthropogenic Drivers of Mammalian Functional Diversity in Bénoué National Park, Cameroon

**DOI:** 10.1002/ece3.72363

**Published:** 2025-10-19

**Authors:** Murielle Majiteu, Simon A. Tamungang, Jan Riegert

**Affiliations:** ^1^ Research Unit for Applied Biology and Ecology, Faculty of Science University of Dschang Dschang West Region Cameroon; ^2^ Biology Department Calvin University Grand Rapids Michigan USA; ^3^ Department of Zoology, Faculty of Science University of South Bohemia České Budějovice Czech Republic

**Keywords:** Bénoué National Park, ecological filtering, functional diversity, human disturbance, mammals

## Abstract

This study investigates the drivers of mammalian functional diversity in Benoué National Park, Cameroon, focusing on the relative roles of environmental filtering, spatial structure, and plant–animal interactions. Using trait‐based approaches, we partitioned the effect of local environmental variables (plant species or land use) and spatial predictors on mammalian functional traits' structure. Based on the first analysis, the presence of tree species as environmental variables highlighted the dual role of vegetation structure as well as the importance of the spatial pattern. Key plant species such as *Burkea africana*, *Gardenia aqualla*, and *Annona senegalensis* were significantly associated with specific functional traits (carnivores, herbivores, and migratory species). According to the second analysis, with land use proportions as environmental variables, the results highlighted strong trait–environment correlations, particularly in response to anthropogenic disturbance and habitat heterogeneity. Species less frequently observed in areas affected by human activities included Lion *Panthera leo* , Giraffe *Giraffa camelopardalis* , and Giant eland *Taurotragus derbianus gigas* . On the other hand, some species did not avoid the areas with increased human activity (Kob *Kobus kob* or Olive baboon *Papio anubis* ). Conservation strategies should integrate vegetation management, landscape‐scale planning, and spatial connectivity to preserve both taxonomic and functional diversity. Our study emphasizes the ecological importance of plant–mammal interactions in tropical savannah.

## Graphical abstract

This study explores the drivers of mammalian functional diversity in Benoué National Park, Cameroon, focusing on the combined effects of environmental filtering, spatial structure, and plant–animal interactions. Results show that both vegetation characteristics and spatial configuration significantly influence trait distribution, highlighting the importance of integrating landscape and plant‐based conservation strategies in tropical savannah ecosystems.

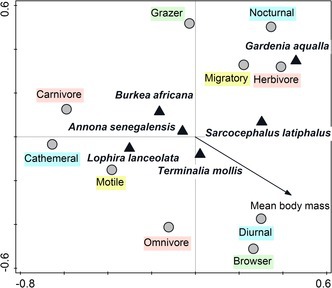

## 1 Introduction

Understanding the processes that shape animal community structure is central to ecology, particularly in systems facing rapid environmental change. In recent years, ecologists have increasingly emphasized the role of functional diversity, the home range, and distribution of species' ecological traits as a key dimension linking biodiversity to ecosystem functioning and resilience (Cardillo et al. 2008; Burton et al. 2011; Mouillot et al. 2013). Functional traits, such as body size, diet, reproductive strategy, activity pattern, and social behavior govern how species interact with their environment and contribute to ecological processes such as nutrient cycling, energy transfer, and population regulation (Isselin 2014; Bison 2015). Thus, identifying the environmental and spatial drivers that influence the distribution of these traits is critical for informing conservation strategies aimed at maintaining ecosystem function.

In tropical savannahs such as those of northern Cameroon, functional diversity among large‐ and medium‐sized mammals is shaped by seasonal climate variability, spatial habitat heterogeneity, and increasing anthropogenic disturbance (Leibold et al. 2004; Cardillo et al. 2008). Environmental filters such as vegetation structure, water availability, and land use intensity select for species with traits adapted to specific conditions such as generalist diets or nocturnal activity. Simultaneously, spatial constraints including habitat fragmentation, dispersal limitation, and land‐use legacies generate nonrandom patterns of species and traits across the landscape (Garrouj 2019; Mollier 2023). These interacting processes can be understood using a meta‐community framework, which integrates species–environment relationships with spatial dynamics (Azihou et al. 2013; Newbold et al. 2020).

In the context of Benoué National Park (BNP) located within the Sudanian savannah zone, these processes operate along complex gradients of disturbance and vegetation. BNP supports a diverse assemblage of medium and large mammals, including herbivores like Kob ( *Kobus kob* ), carnivores like lion ( *Panthera leo* ), and omnivores such as baboons ( *Papio anubis* ) that differ markedly in their ecological traits. However, the park is exposed to poaching, agricultural expansion, livestock grazing, and settlement encroachment (Angwafo 2006; Valeix 2020; Hamadou et al. 2021; Seibou et al. 2021). These anthropogenic pressures act as environmental filters that may differentially affect species depending on their traits; for example, favoring generalist, mobile, or nocturnal species while excluding more specialized or disturbance‐sensitive taxa (Gaynor et al. 2018; Newbold et al. 2020).

Plant species composition and structure also exert a strong effect on mammal communities by determining both resource availability and habitat complexity. In savannah systems, plants not only provide food resources for foraging but also define the structural characteristics of habitats that influence species' behavior, movement, and survival (Dohn et al. 2013; Estes et al. 2013). Some plant species may act as keystone or foundation species, supporting a wide array of ecological interactions and promoting overall functional diversity. Therefore, vegetation structure acts as both a direct resource and an ecological filter that mediates the presence and persistence of particular functional traits within animal communities. Loss of these plant species, especially those with foundational or keystone roles, can therefore produce cascading changes in mammalian functional diversity (Bulus et al. 2011; Prato et al. 2013).

Despite the importance of these ecological processes, few studies have explicitly examined the combined influence of environmental conditions, spatial structure, and plant species composition on mammalian functional diversity in African savannah landscapes (Vandewalle et al. 2010; Azihou et al. 2013; Rubio and Swenson 2022; Liu et al. 2023). This study addresses that gap by analyzing the functional trait distribution of mammal species such as mean body mass, trophic guild, diet, social behavior, migratory status, and activity period across BNP. Specifically, we aim to (1) quantify the relative contributions of environmental filtering and spatial processes in shaping the distribution of functional traits among mammals, (2) assess the influence of key plant species in mediating mammal–trait habitat relationships and structuring functional diversity, and (3) evaluate the ecological and conservation implications of these patterns under current anthropogenic pressures. By integrating functional trait data with vegetation and spatial modeling, this research advances understanding of plant–animal interactions, trait‐based assembly processes, and biodiversity management in fragmented savannah ecosystems. It also provides a framework for targeting conservation efforts toward maintaining ecosystem function and resilience, not merely species richness.

## 2 Methods

### 2.1 Study Area

**FIGURE 1:**
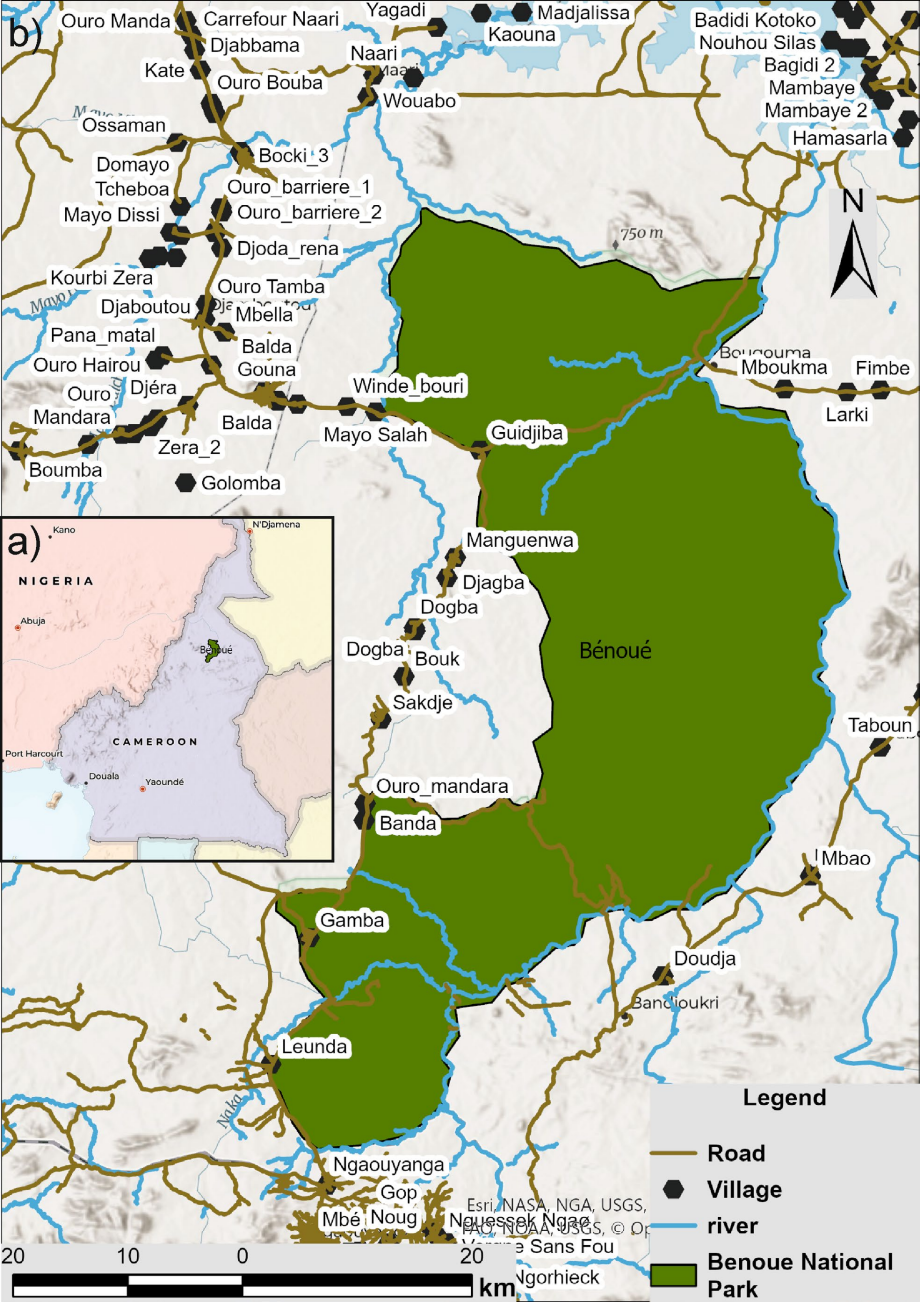
Map of (a) location of study area within Cameroon and (b) Bénoué National Park. ESRI topography basemap was used to create the figure.

Bénoué National Park (Figure 1) was established in 1968 and designated a UNESCO Biosphere Reserve in 1981. The National Park is located in the North Region of Cameroon between the towns of Garoua and Ngaoundéré, spans within an area of approximately 1800 km^2^ (180,000 ha), and lies within the Bénoué Department. Its mean geographical coordinates are approximately 8.3468° N and 13.8744° E, with elevations ranging from 250 to 760 m a.s.l. The area is characterized by a diverse landscape of wooded grasslands, savannahs, and rocky massifs. It is bordered to the east by the Bénoué River, which stretches over 100 km along the park's boundary, providing a vital water source for the area's flora and fauna. The vegetation is predominantly Guinea–Sudan savanna, featuring *Isoberlinia doka* woodland and *Terminalia macroptera* open savanna, with grass species such as *Hyparrhenia*, *Andropogon*, and *Loudetia*.

Bénoué National Park supports a rich biodiversity, including approximately 17 species of ungulates like Kob, Western hartebeest ( *Alcelaphus buselaphus* ), Giant eland ( *Taurotragus derbianus* ), and Waterbuck ( *Kobus ellipsiprymnus* ). Other notable wildlife includes African elephant ( *Loxodonta africana* ), Spotted hyena ( *Crocuta crocuta* ), Warthog ( *Phacochoerus africanus* ), and various monkey species. The park is also an Important Bird Area, with over 300 bird species recorded, being a significant site for avian diversity.

The climate in the park is characterized by a wet season from May to September and a dry season from October to April, with annual rainfall ranging between 1200 and 1500 mm. During the dry season of 2003–2004, approximately 85% of the park was subjected to controlled burns by park management to facilitate game viewing and stimulate regrowth for herbivores. Access to Bénoué National Park is primarily via the main road linking Garoua and Ngaoundéré, with the public road to Tcholliré cutting across the northern part of the park.

### 2.2 Data Collection

#### 2.2.1 Mammal Distribution Survey

Data were collected during the dry season, between February and May 2024 in Bénoué National Park. The guided reconnaissance (recce) method was used for surveying mammals and recording human activities (Mouzoun 2018; Segniagbeto et al. 2022; Lyngdoh et al. 2023). Using ArcGIS Pro software (ESRI 2011), 20 sampling points with a 3.5 km radius were randomly distributed across the entire park, covering various vegetation types. The total area of all buffer zones represented approximately 43% of the park's total surface area (770 km^2^ out of 1800 km^2^). The study was conducted within these buffer zones as follows: the center of each point was selected as the starting location. Each morning, we began a reconnaissance walk following a straight line in a randomly chosen direction, with an average distance of 3.5 km. Then, we changed direction for approximately 2 km to take a second 3.5 km line leading back to the starting point, forming an isosceles triangle‐shaped trajectory. In total, we covered an average distance of 9 km per day over a 5‐day period per sampling point. The walk followed these predefined paths at a low speed (approximately 1 km/h) to conduct both direct (animal sightings) and indirect (signs of presence such as droppings, footprints, feeding traces, etc.) observations of wildlife, as well as signs of human activity (machete cuts, footprints, bullet casings, signs of transhumance, poaching, agricultural sites, mining sites, etc.). Recorded observations were located at a distance of 5–10 m on either side of the walking path. All surveys were conducted during daylight hours, between 8:00 a.m. and 6:00 p.m. For each detection, the following information was recorded: time of observation, type of sign, number of signs observed, species, soil type (sandy, clayey, or rocky), and geographic coordinates recorded using a Garmin 60csx GPS. Vegetation data were collected as follows: during the patrols, for each recorded wildlife observation, we estimated the percentage cover of grasses, shrubs, and trees within a 50‐m radius around the observation site. In addition, using binoculars, dominant shrub and tree species were also identified guiding by Arbonnier's (2019) plant identification guide.

### 2.3 Remote Sensing Datasets

To assess the effect of habitat characteristics on mammal distribution, several environmental variables were extracted from satellite data covering the data collection period. The satellite images were integrated into ArcGIS Pro software (ESRI 2011) and associated with the buffers around species observations. The environmental parameters retained were as follows:

1. Distance to nearest village (km) calculated from the GPS coordinates of observation points, using the GIS's Euclidean distance function to measure the distance to the nearest village.
2. Mean human population size within each buffer based on GHS‐POP database (Schiavina et al. 2023).
3. Mean tree height within each buffer based on Global Forest Canopy Height 2019 database (Potapov et al. 2020).
4. Mean tree cover within each buffer based on Global Forest Watch database (Hansen et al. 2013).
5. Land cover type obtained by supervised classification of high‐resolution satellite images, with field verification to validate spectral signatures based on ESACCI‐LC‐L4‐LC10‐Map‐20 m database (Ramoino et al. 2018).
6. Normalized Difference Vegetation Index (NDVI) is used as an indicator of plant biomass and primary productivity. NDVI was derived from satellite images, depending on data availability and cloud cover conditions (Living Atlas in ArcGIS Pro—Sentinel 2).
7. Elevation (m a.s.l.) extracted from a Digital Elevation Model (DEM) from the Shuttle Radar Topography Mission (SRTM), with a spatial resolution of 30 m (ALOS World 3D—30 m, AW3D30; Takaku et al. 2020).

All these variables were standardized before inclusion into the multivariate statistical analysis to facilitate interpretation and comparison.

### 2.4 Functional Traits

We obtained data on mammalian functional traits through a literature search (Weiss and Ray 2019; Gorczynski et al. 2021). Each monitored mammalian species was assigned ranked values for the following functional traits: mean body mass (kg), trophic guild (herbivore, carnivore, and omnivore), diet (browser, carnivore, and grazer), social behavior (social and solitary), migratory status (motile, migratory, and sedentary), and activity period (diurnal, nocturnal, and cathemeral, Table S1). Additionally, the conservation status of species was determined based on the International Union for Conservation of Nature (IUCN 2022) Red List.

### 2.5 Statistical Analyses

The intercorrelations between environmental variables and functional traits were analyzed using variance partitioning by principal coordinate analysis of neighbor matrices (PCNM) in Canoco 5 software (ter Braak and Šmilauer 2012), which was recommended by Marrot et al. (2015). This multivariate analysis enabled us to remove the effect of geographical position (i.e., space predictors) from the effect of primary predictors (Legendre and Legendre 2012). The analysis is suitable for calculating intercorrelated variables since all these variables enter the analysis simultaneously. The analysis included nine steps: (1) primary predictor test (i.e., preliminary test of the overall effect of primary predictors on the dataset), (2) primary predictor testing by partial canonical correspondence analysis (CCA) based on partial Monte‐Carlo permutation tests (*n* = 499 permutations), (3) principal coordinate analysis (PCoA) based on Euclidean distances (i.e., finding the main space predictors based on GPS coordinates), (4) PCNM for all predictors (i.e., preliminary test of the overall effect of space predictors on the dataset), (5) PCNM selection (i.e., the choice of space predictors based on coordinates using forward selection and partial Monte‐Carlo permutation tests), (6) spatial effects analysis (i.e., assessing the amount of variability explained by space predictors), (7) primary predictor effects analysis (i.e., assessing the amount of variability explained by primary predictors), (8) joint effects analysis (i.e., assessing the amount of variability explained by both predictor types), and (9) removal of spatial effects (Šmilauer and Lepš 2014). Firstly, we prepared the community weighted means table for further comparisons using PCNM. In the analyses, we used these functional traits: litter size (*n*), migratory status (motile, sedentary, migratory, and solitary), social behavior (social and solitary), trophic guild (herbivore, omnivore, and carnivore), diet (browser, grazer, frugivore, lignivore, and carnivore), substrate use (terrestrial and arboreal), activity period (diurnal, nocturnal, and cathemeral), and IUCN Red List category (least concern, near threatened, and vulnerable).

We performed two PCNM analyses with different environmental variables. For the first analysis, we used the presence/absence of each tree species (*Burkea africana*, *Sarcocephalus latiphalus*, *Gardenia aqualla*, *Terminalia mollis*, *Annona senegalensis* , *Lophira lanceolata*). For the second analysis, we used soil type (clay, stony, and sandy), human population size (*n*), mean tree height (m), distance to river (km), tree cover (%), shrub land (%), grassland (%), built‐up area (%), open water (%), cropland (%), bare land (%), mean NDVI, and mean buffer elevation within a buffer of each mammal species based on Broekman et al. (2023) around each count point as independent variables (i.e., predictors) in the PCNM analysis; a data unit was one field observation (*n* = 528). The home range sizes for each species are available in Table S2.

## 3 Results

### 3.1 Recorded Species

We documented the presence of 20 mammal species within BNP, spanning four taxonomic orders: *Artiodactyla*, *Carnivora*, *Primates*, and *Rodentia*. These species were distributed across nine families, with Bovidae being the most frequently observed (453 records), followed by Cercopithecidae (18), Hyaenidae (18), Suidae (16), Canidae (8), Giraffidae (8), Viverridae (5), Felidae (1), and Hystricidae (1).

### 3.2 Functional Traits and Trees Species

**FIGURE 2:**
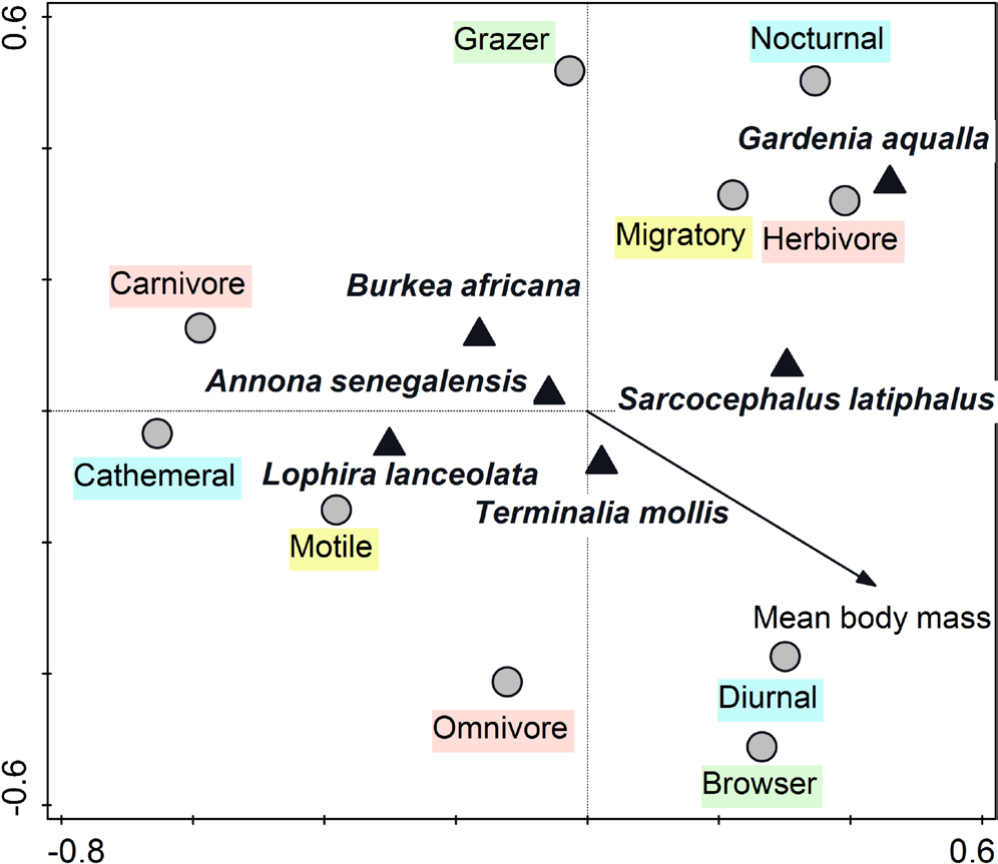
PCNM ordination diagram of mammal species functional traits and presence of main tree species in Bénoué National Park, Cameroon (*n* = 528 observations). First and second ordination axes together explained 42.5% of variability. The functional trait variables are represented by circles or small arrow, the bigger triangular dot represents plant species variables (*Burkea africana*, *Sarcocephalus latiphalus*, *Gardenia aqualla*, *Terminalia mollis*, *Annona senegalensis* , *Lophira lanceolata*).

Based on PCNM analysis, the plant species variables explained 42.5% of variability, space predictors explained 39.5% of variability, and the shared fraction was 22.2% of variability. We found a statistically significant effect of five plant species (Table 1). The presence of *B. africana* (*r*_1_ = −0.28), *A. senegalensis* (*r*_1_ = −0.25), and *L. lanceolata* (*r*_1_ = −0.26) was negatively correlated with the first ordination axis. The presence of *T. mollis* (*r*_2_ = −0.21) was negatively correlated with the second ordination axis. The presence of *G. aqualla* was positively correlated with the first (*r*_1_ = 0.22) and second (*r*_2_ = 0.17) ordination axes. We also found a significant effect of the presence of plant species variables on the functional traits of mammals. There was a clear gradient between all functional trait variables along the ordination axes. The presence of carnivore species was negatively correlated with the first (*r*_1_ = −0.62) and positively correlated with the second (*r*_2_ = 0.23) ordination axes. On the other hand, herbivore species were positively correlated with the first (*r*_1_ = 0.42) and second (*r*_2_ = 0.35) ordination axes. Omnivore species were negatively correlated with the first (*r*_1_ = −0.22) and second (*r*_2_ = −0.43) ordination axes. Grazer species were positively correlated with the second (*r*_2_ = 0.52) ordination axis. Browser species were positively (*r*_1_ = 0.28) and negatively (*r*_2_ = −0.52) correlated with the first and second ordination axes, respectively. Migratory species were positively correlated with the first (*r*_1_ = 0.22) and second (*r*_2_ = 0.36) ordination axes. On the other hand, motile species were negatively correlated with the first (*r*_1_ = −0.38) and second (*r*_2_ = −0.25) ordination axes. Body mass was positively and negatively correlated with the first (*r*_1_ = 0.25) and second (*r*_2_ = −0.28) ordination axes, respectively. The presence of diurnal species was positively correlated with the first (*r*_1_ = 0.35) and negatively correlated with the second (*r*_2_ = −0.38) ordination axis. Cathemeral species were negatively correlated with the first (*r*_1_ = −0.67) ordination axis; nocturnal species were positively correlated with the first (*r*_1_ = 0.37) and second (*r*_2_ = 0.52) ordination axes (Figure 2).

**TABLE 1:** The results of the PCNM analysis on the effect of plant species occurrence on the mammal community structure based on functional traits. I and II ordination axes together explained 42.5% of variability. PCO—space predictor.

Tree species	Contribution %	Pseudo‐*F*	*p*
*Lophira lanceolata*	6.27	4.8	0.002
*Burkea africana*	5.86	4.5	0.002
*Sarcocephalus latiphalus*	6.42	4.9	0.002
*Gardenia aqualla*	4.99	3.8	0.002
*Terminalia mollis*	3.38	2.9	0.014
*Annona senegalensis*	2.35	2.7	0.062
PCO.3	6.71	9.2	0.002
PCO.59	3.91	5.5	0.002
PCO.20	3.72	5.2	0.002

Thus, species with carnivore (Lion) and grazer (Kob) functional traits preferred the presence of *B. africana*, while migratory (African buffalo *Syncerus caffer* ), nocturnal species (African civet *Civettictis civetta* ) as well as herbivores (Bushbuck *Tragelaphus scriptus* ) were found within the sites with the presence of *G. aqualla* and *S. latiphalus* trees. In addition, *T. mollis* and *A. senegalensis* were attractive tree species for all the species. *L. lanceolata* was correlated with the presence of cathemeral and motile species (Kob, Oribi *Ourebia ourebi* , Figure 2).

### 3.3 Effect of Environmental Variables on Mammals' Functional Traits

The PCNM analysis revealed strong spatial and environmental structuring of mammalian functional traits in BNP. The variation partitioning analysis indicated that environmental variables explained 49.3% of the variability in mammal functional community structure, while spatial variables accounted for 48.3%, and 6.2% was the shared fraction. These results underline the dual influence of both environmental filtering and spatial processes, such as dispersal limitation, on mammalian community structure (Table 2).

**TABLE 2:** The results of the PCNM analysis on the effect of environmental variables on the mammal community structure based on functional traits. I and II ordination axes together explained 51.5% of variability. PCO—space predictor.

Environmental variable	Contribution %	Pseudo‐*F*	*p*
Bare area (%)	12.25	8.6	0.002
Grassland (%)	8.25	6.3	0.004
Cropland (%)	7.41	5.9	0.036
Distance to village (km)	3.65	2.8	0.010
Tree height (m)	4.16	2.6	0.018
NDVI	4.12	2.5	0.022
PCO.3	6.72	9.2	0.002
PCO.59	3.98	5.9	0.002
PCO.20	3.75	5.3	0.002

**FIGURE 3:**
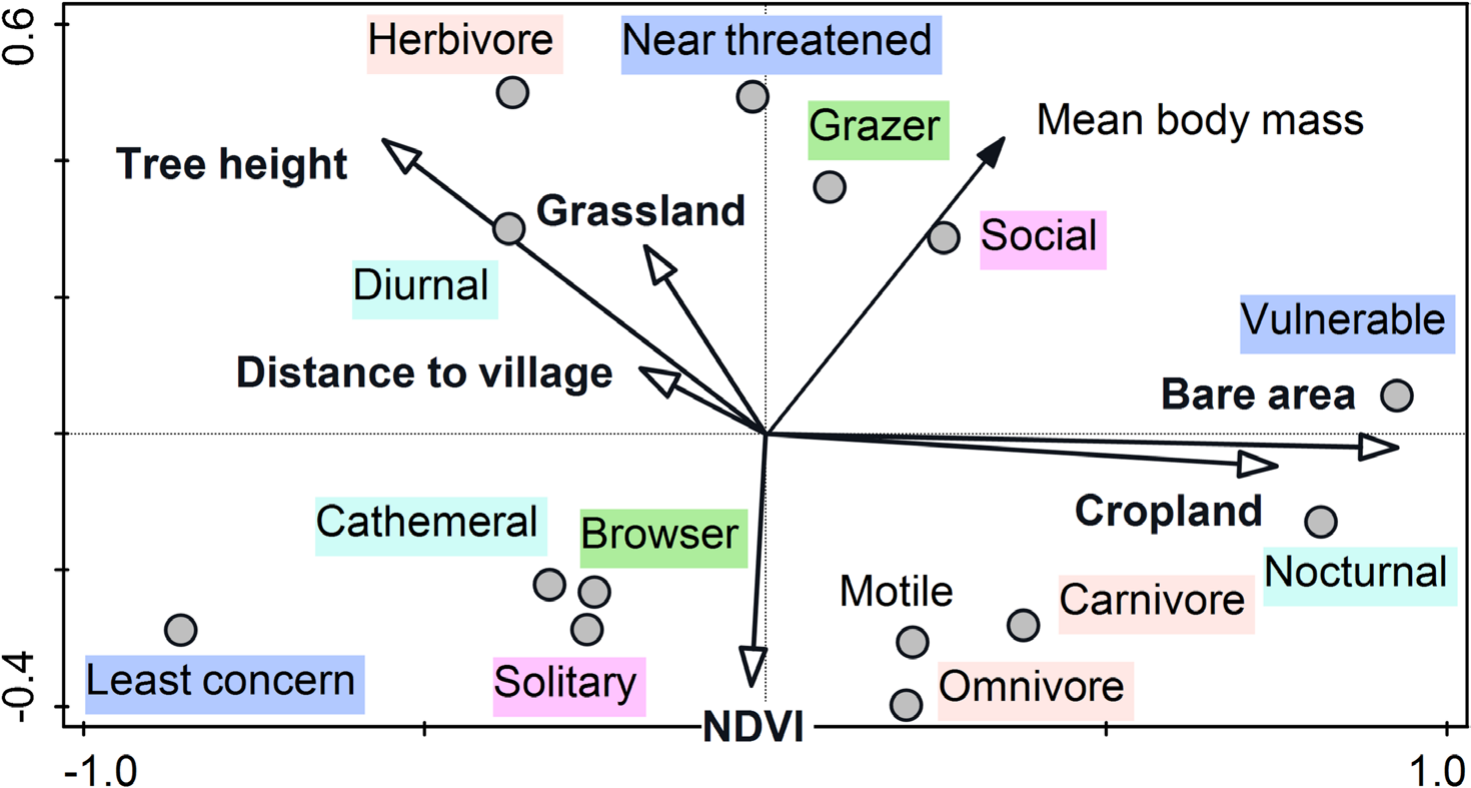
PCNM ordination diagram of mammal species functional traits and environmental factors in Bénoué National Park, Cameroon (*n* = 528 observations). First and second ordination axes together explained 51.5% of variability. The functional traits variables are represented by circles or small arrows; the bigger arrows represent environmental variables (grassland, cropland, bare area, tree height, NDVI, and distance to village).

The first two PCNM axes together explained a substantial proportion of the variability in functional trait distributions, revealing nonrandom patterns in trait assemblages. The first ordination axis corresponded to a gradient of increasing urbanization and human impact. We found a positive correlation of bare area (*r*_1_ = 0.36) and cropland (*r*_1_ = 0.28) and a negative correlation of tree height (*r*_1_ = −0.22) with the first ordination axis, indicating that areas on the left of the axis are less disturbed and more forested. The second axis reflected distance from human settlements. Distance to village was positively correlated with the second axis (*r*_2_ = 0.36) and similar correlations were found for proportion of grassland (*r*_2_ = 0.26). NDVI was negatively correlated with the second ordination axis (*r*_2_ = −0.31). Our results suggest that sites further from human activity were typically dominated by shrubland and grassland harboring mammal communities with distinct functional traits (Figure 3).

The functional traits were aligned along these environmental gradients. The direction and strength of correlations provided ecological insight into how mammalian life‐history strategies respond to habitat structure. Some traits were positively correlated with urbanized or disturbed areas (right side of the first axis), including nocturnal species (*r*_1_ = 0.83), vulnerable species (*r*_1_ = 0.95), carnivores (*r*_1_ = 0.32), motile species (*r*_1_ = 0.27), and omnivores (*r*_1_ = 0.24). These species may exploit human‐altered landscapes or avoid humans by being active at night. Traits negatively correlated with the first ordination axis (i.e., natural habitats) included cathemeral (*r*_1_ = −0.34), least concern species (*r*_1_ = −0.87), solitary species (*r*_1_ = −0.28), browsers (*r*_1_ = −0.25), and herbivores (*r*_1_ = −0.39). These species relied on intact habitats with low disturbance. The second axis (distance from village) showed positive correlations for near threatened species (*r*_2_ = 0.55), herbivores (*r*_2_ = 0.52), diurnal species (*r*_2_ = 0.32), and grazers (*r*_2_ = 0.38). This result indicated that areas distant from villages support more threatened, day‐active, and grass‐eating species, reflecting low anthropogenic pressure. Conversely, traits like carnivory (*r*_2_ = −0.38), nocturnality (*r*_2_ = −0.23), and omnivory (*r*_2_ = −0.45) were negatively correlated with the second ordination axis, suggesting that these species were more prevalent close to human‐altered environments (Figure 3).

The ordination highlighted distinct trait‐based community assemblages structured by human disturbance, habitat type, and spatial location. Mammals in more remote and less disturbed environments tended to be diurnal, herbivorous, and less motile, while those near disturbed areas are more likely to be nocturnal, omnivorous, or carnivorous, and vulnerable.

## 4 Discussion

The identification of 20 mammal species across four taxonomic orders and nine families in BNP reflects a moderate level of species richness for a savannah ecosystem under increasing anthropogenic pressure. The dominance of the Bovidae family, comprising over 90% of all recorded observations, is consistent with the park's savannah‐dominated landscape, which supports a wide range of grazers and browsers adapted to open habitats. This functional group plays a critical role in ecosystem processes such as vegetation control and nutrient cycling (Garrouj 2019; Valeix 2020; Rubio and Swenson 2022; Mollier 2023). However, these findings must be interpreted in light of several methodological limitations that could have influenced species detectability and thus biased the richness estimates.

First, the observation method, based on guided recce walks, inherently favors the detection of species that are diurnal, terrestrial, and relatively tolerant of human presence. Mammals with cryptic behaviors, arboreal habits, or strictly nocturnal activity patterns are less likely to be observed directly, especially during surveys conducted only in daylight hours. While indirect signs (e.g., tracks, dung, feeding traces) were used to supplement direct observations, the reliability of such signs varies by species and may be difficult to attribute unambiguously without camera trap confirmation or DNA‐based techniques (Mouzoun 2018). Some species may leave few detectable traces or share similar signs with others, introducing a risk of under‐ or overestimation of their presence. Second, the timing of data collection corresponds to the dry season in the Sudanian savannah, when water and resource availability is more limited. This seasonal window may have influenced mammal distribution and behavior, potentially increasing visibility and encounter rates for some species near water sources, while causing others to migrate temporarily outside the sampling areas (Hamadou et al. 2021). Therefore, the seasonality of the survey may not capture the full spectrum of species using the park year‐round, especially migratory or seasonally nomadic taxa (Angwafo 2006). Third, the spatial design, although randomized and covering 43% of the park's surface, may not have fully captured all habitat types or microhabitats, particularly those more remote or inaccessible. As presented by Devictor and Robert (2009), this could lead to a spatial sampling bias, excluding species that are habitat specialists or restricted to less‐represented vegetation types.

In summary, while the results provide valuable insights into community structure and functional traits, they represent a part of the park's mammal diversity. Future studies can benefit from integrating complementary methods such as camera traps, acoustic monitoring, or environmental DNA (eDNA), and from conducting surveys across multiple seasons to obtain a more comprehensive and unbiased picture of mammal diversity and distribution patterns in Bénoué National Park.

### 4.1 Presence of Tree Species and Functional Traits

Our results highlight the key role of plant species and spatial factors in structuring mammalian functional traits, confirming and enriching current knowledge on plant–mammal interactions. This partition highlights the complexity of factors governing the functional structuring of animal communities (Garrouj 2019; Valeix 2020; Mollier 2023). The strong spatial component highlights dispersal constraints or historical effects, while the plant part confirms the importance of vegetation as an ecological filter. The shared fraction indicates that plants were not only resources for mammals but also indicators of spatial heterogeneity, as their distribution was affected by geographic or edaphic factors.

The specific associations between plant species and mammalian functional traits highlight the central role of plants in ecosystem dynamics. For example, the presence of *B. africana* is associated with the presence of both carnivores (Spotted hyena, Lion, etc.) and herbivores (e.g., Hartebeest and Giant eland). This association could be related to the ability of this plant species to provide shelter or hunting ground for carnivores and food resources for herbivores, as suggested by studies on tropical forest habitats (Shorrocks and Bates 2014; Naah 2020). *G. aqualla* and *S. latiphalus* attract migratory species (African buffalo, Giraffe, Kob, etc.), nocturnal species (African civet, Side‐striped jackal *Lupulella adustus*, Lion), and herbivorous species, probably due to the availability of fruits or other nutritional resources, which is a well‐documented phenomenon within tropical areas (Da et al. 2023; Alarape et al. 2024). *T. mollis* and *A. senegalensis* appear to promote general functional diversity, which may reflect their role as foundation species in savannas, providing a range of ecological services (Bulus et al. 2011; Dohn et al. 2013; Dohn 2015).

The gradients observed for mammalian functional traits were consistent with the dynamics of species adaptation to environmental conditions. For example, carnivores had a negative and positive correlation with the first and second ordination axes, respectively, reflecting their dependence on specific habitats favoring hunting, often associated with particular plant resources that attract their prey (Isselin 2014). Herbivores and migrants were positively correlated with both the ordination axes, indicating a flexible use of habitats rich in plant species. This is consistent with studies showing that generalist herbivores adapt to complex landscapes (Fryxell et al. 2008). Omnivores (Tantalus monkey *Chlorocebus tantalus* , Guereza *Colobus guereza* , Common duiker *Sylvicapra grimmia* , Common warthog, etc.), having a weak association with the ordination axes, could indicate a lesser dependence on specific plant species, as has been reported for opportunistic omnivorous species (McLaren et al. 2021). For grazers (Hartebeest, Roan antelope, African buffalo, etc.) and browsers (Giraffe, Bushbuck, Common duiker, Giant eland, etc.), the distinct correlations with axes reflect their functional specialization in plant resource use, an observation that has implications for rangeland and savannah management (Bison 2015).

### 4.2 Environmental Factors and Functional Diversity

Our study highlighted the importance of both environmental filtering and spatial processes in structuring mammalian functional diversity within Bénoué National Park. These findings suggested that both niche‐based mechanisms and dispersal limitation played substantial roles in determining community structure, consistent with meta‐community theory (Garrouj 2019; Valeix 2020; Mollier 2023).

The significant relationship between mammal functional traits and environmental gradients reflected the influence of habitat heterogeneity and human disturbance on species distributions. The positive correlation of urbanized land as cropland with the first ordination axis, contrasted with the negative association of tree height, implies that anthropogenic land‐use change is a key driver of functional turnover. Similar patterns have been documented in African savanna ecosystems, where habitat degradation and fragmentation selectively filter species based on body size, trophic guild, and behavioral flexibility (Newbold et al. 2020; Liu et al. 2023).

Functional traits associated with large body size (Giraffe, African buffalo, Giant eland, etc.), carnivory species (Spotted Hyena), and species vulnerable to extinction (Lion, Giraffe, Giant eland) were positively aligned with urban and disturbed environments. While this may seem counterintuitive, these species are often more mobile and capable of avoiding human presence through temporal niche shifts, such as increased nocturnal behavior (Gaynor et al. 2018). However, their presence in such habitats may also reflect edge effects or proximity to protected core areas. Conversely, functional traits such as cathemerality, solitary behavior, and browsing were negatively correlated with disturbance, indicating that these species are more dependent on intact and less disturbed habitats. This aligns with studies indicating that browsers are more vulnerable to habitat structure changes due to their reliance on woody vegetation (Linksvayer and Janssen 2009; Sol et al. 2013; Sundrum 2015; Rowan and Faith 2019).

The second ordination axis primarily captured the effect of distance from human settlements, reinforcing the role of anthropogenic influence on trait distribution. Diurnal, grazing, and herbivorous species were more abundant within areas far from villages, suggesting that they are more susceptible to human activity, potentially due to hunting or other disturbances (Ripple et al. 2017). The negative association of carnivores and omnivores with this axis further suggests that these groups may exploit human‐modified landscapes, either opportunistically or due to increased prey availability (Brearley et al. 2013; Sol et al. 2013). The identification of nonrandom patterns in trait‐environment relationships supports the environmental filtering hypothesis, whereby environmental conditions constrain the range of traits that can persist locally (Linksvayer and Janssen 2009; Sundrum 2015). The comparable influence of spatial variables highlights the importance of non‐environmental constraints such as limited dispersal processes, past disturbances, or regionally structured dynamics in shaping mammal communities. This suggests that community assembly is not solely a response to current environmental filtering, but also reflects the imprint of spatial processes like habitat connectivity, fire history, or predator–prey interactions, which may operate at broader temporal and spatial scales (Sol et al. 2013). These findings underscore the need to consider landscape configuration and historical land use when designing conservation interventions, as functional diversity may persist or erode depending on species' ability to move across fragmented habitats.

Importantly, these results underscore the functional consequences of land‐use change. Changes in the spatial distribution of functional traits can affect ecosystem functioning and resilience, especially in tropical ecosystems where functional redundancy may be limited (Newbold et al. 2020). The loss or displacement of species with key ecological roles (e.g., large herbivores or apex predators) may lead to cascading effects on vegetation structure, nutrient cycling, and trophic interactions (Gaiballa and Lee 2012; Estes et al. 2013). Given that spatial and environmental factors jointly affect functional diversity, conservation strategies in BNP should adopt a landscape‐scale approach maintaining a mosaic of habitats and minimizing habitat degradation near human settlements. Buffer zones, improved land‐use planning, and anti‐poaching efforts around high‐diversity zones can help preserve both taxonomic and functional diversity.

### 4.3 Ecological Implications and Conservation

Mammalian functional diversity in Bénoué National Park was affected by the composition and spatial distribution of plant species. The presence of certain plant species, such as *B. africana* and *G. aqualla*, played a central role in supporting different mammalian functional groups by providing food, shade, cover, or breeding habitats. These species appear to act as functional keystone plants, structuring the availability of ecological niches across the landscape. Their loss or decline could disproportionately affect mammal communities, especially specialist or habitat‐dependent species. This reinforces the idea that plant diversity conservation is not only vital for maintaining floristic richness but also for sustaining ecosystem processes that depend on cross‐taxa interactions (Estes et al. 2013; Prato et al. 2013; Badjaré et al. 2018). To mitigate risks of functional erosion, conservation efforts should prioritize the protection and regeneration of key woody species that support high mammalian functional diversity. Management plans should identify tree and shrub species that are strongly associated with functionally important mammal groups (e.g., large herbivores, frugivores, or nocturnal omnivores) and promote their natural regeneration in degraded areas. In particular, areas where *B. africana* and *G*. *aqualla* are dominant should be considered for strict protection status or restoration interventions, as these habitats appear to act as ecological hotspots for trait diversity.

Moreover, the observed correlations between functional traits and environmental gradients suggest that changes in vegetation structure caused by deforestation, bushfires, or climate variability could rapidly disrupt existing plant–mammal associations (Cardillo et al. 2008; He et al. 2019; Adla et al. 2022; Sharma and Birman 2024). Conservation strategies should therefore incorporate fire management plans, anti‐deforestation measures, and climate adaptation strategies focused on maintaining habitat heterogeneity and connectivity. This could include establishing buffer zones around ecologically sensitive habitats, enhancing natural corridors between fragmented forest patches, and limiting extractive activities in plant‐rich areas.

Finally, integrating plant functional roles into monitoring and management frameworks would allow protected area managers to track early signs of functional collapse. Monitoring programs should not only record species presence or abundance but also consider the trait profiles of both plants and animals, particularly in zones under strong anthropogenic pressure. Conservation policy in savannah ecosystems like BNP must evolve beyond species‐focused approaches to embrace trait‐based and interaction‐based indicators of ecosystem health. Protecting the mutual dependencies between plants and mammals will be essential to maintaining ecosystem resilience and ensuring long‐term biodiversity conservation in the region.

## Author Contributions

**Murielle Majiteu:** conceptualization (equal), data curation (equal), formal analysis (equal), methodology (equal), project administration (equal), resources (equal), validation (equal), visualization (equal), writing – original draft (equal), writing – review and editing (lead). **Simon A. Tamungang:** conceptualization (equal), supervision (equal), visualization (equal), writing – review and editing (equal). **Jan Riegert:** conceptualization (equal), data curation (equal), formal analysis (equal), methodology (equal), software (equal), supervision (equal), validation (equal), writing – review and editing (equal).

## Conflicts of Interest

The authors declare no conflicts of interest.

## Supporting information


**Data S1:** Environmental data and functional traits.

**Table S1:** Overview of functional traits of the species in the Benoue National Park, Cameroon. Functional traits include feeding type, body mass, and activity pattern based on literature review and number of observations for group size.
**Table S2:** Home range sizes of recorded mammal species according to Broekman et al. (2022).

## Data Availability

Data are available in Data S1.
