# Supplementary material for: Environmental and Anthropogenic Drivers of Mammalian Functional Diversity in Bénoué National Park, Cameroon
Source: Ecol Evol. 2025 Oct 19;15(10):e72363. doi: 10.1002/ece3.72363 (PMC12535917; doi:10.1002/ece3.72363)
Supplement: Supplementary file 2 — Table S1: Overview of functional traits of the species in the Benoue National Park, Cameroon. Functional traits include feeding type, body mass, and activity pattern based on literature review and number of observations for group size. Table S2: Home range sizes of recorded mammal species according to Broekman et al. (2022). [file ECE3-15-e72363-s001.docx]

# Supplementary material

# Environmental and anthropogenic drivers of mammalian functional diversity in Bénoué National Park, Cameroon

Ecology & Evolution

Murielle Majiteu^1*^, Simon A. Tamungang^1, 2^, Jan Riegert^3^

^1^Research Unit for Applied Biology and Ecology, Faculty of Science, University of Dschang, West Region, Cameroon

^2^Biology Department, Calvin University, Grand Rapids, Michigan, United States of America

^3^Department of Zoology, Faculty of Science, University of South Bohemia, Branišovská 1760, 370 05 České Budějovice, Czech Republic

*Corresponding author: Murielle Majiteu, e-mail address: [muriellemajiteu@gmail.com](mailto:muriellemajiteu@gmail.com)

Full postal address: Research Unit for Applied Biology and Ecology, Faculty of Science, University of Dschang, West Region, Cameroon

ORCIDs and e-mails:

Murielle Majiteu: <https://orcid.org/0009-0004-5092-4284>, [muriellemajiteu@gmail.com](mailto:muriellemajiteu@gmail.com)

Simon A. Tamungang: <https://orcid.org/0000-0003-3449-9359>, [atamungang@yahoo.com](mailto:atamungang@yahoo.com)

Jan Riegert: <https://orcid.org/0000-0003-4195-1278>, [honza@riegert.cz](mailto:honza@riegert.cz)

Table S1. Overview of functional traits of the species in the Benoue National Park, Cameroon. Functional traits include feeding type, body mass and activity pattern based on literature review and number of observations for group size.

| Scientific name | Mean body mass | Migratory status | Social behaviour | Trophic guild | Diet | IUCN Red list category | Substrate use | Activity period | N |
| --- | --- | --- | --- | --- | --- | --- | --- | --- | --- |
|  |  |  |  |  |  |  |  |  |  |
| *Alcelaphus buselaphus* | 100 | Sedentary | Social | Herbivore | Grazer | Least Concern | Terrestrial | Diurnal | 89 |
| *Cephalophus rufilatus* | 12 | Sedentary | Solitary | Herbivore | Browser | Least Concern | Terrestrial | Diurnal | 42 |
| *Giraffa camelopardalis* | 1180 | Migratory | Social | Herbivore | Browser | Vulnerable | Terrestrial | Diurnal | 8 |
| *Hippotragus equinus* | 220 | Migratory | Social | Herbivore | Grazer | Vulnerable | Terrestrial | Nocturnal | 92 |
| *Hystrix cristata* | 13 | Sedentary | Solitary | Herbivore | Lignivore | Least Concern | Terrestrial | Nocturnal | 1 |
| *Chlorocebus tantalus* | 8 | Sedentary | Social | Omnivore | Frugivore | Least Concern | Arboreal | Diurnal | 7 |
| *Kobus ellipsiprymnus* | 200 | Migratory | Social | Herbivore | Browser | Least Concern | Terrestrial | Cathemeral | 15 |
| *Kobus kob* | 100 | Migratory | Social | Herbivore | Grazer | Least Concern | Terrestrial | Cathemeral | 92 |
| *Papio anubis* | 20 | Sedentary | Social | Omnivore | Frugivore | Least Concern | Arboreal | Diurnal | 9 |
| *Tragelaphus scriptus* | 60 | Migratory | Solitary | Herbivore | Browser | Least Concern | Terrestrial | Diurnal | 34 |
| *Civettictis civetta* | 13 | Motile | Solitary | Omnivore | Frugivore | Least Concern | Terrestrial | Nocturnal | 5 |
| *Colobus guereza* | 10 | Sedentary | Social | Omnivore | Frugivore | Least Concern | Arboreal | Diurnal | 2 |
| *Crocuta crocuta* | 60 | Sedentary | Social | Carnivore | Carnivore | Least Concern | Terrestrial | Diurnal | 18 |
| *Lupulella adustus* | 10 | Solitary | Social | Omnivore | Carnivore | Least Concern | Terrestrial | Nocturnal | 8 |
| *Ourebia ourebi* | 18 | Motile | Social | Herbivore | Grazer | Least Concern | Terrestrial | Diurnal | 20 |
| *Panthera leo* | 250 | Sedentary | Social | Carnivore | Carnivore | Vulnerable | Terrestrial | Nocturnal | 1 |
| *Phacochoerus africanus* | 100 | Migratory | Social | Omnivore | Grazer | Least Concern | Terrestrial | Diurnal | 16 |
| *Syncerus caffer* | 600 | Migratory | Social | Herbivore | Grazer | Near Threatened | Terrestrial | Diurnal | 24 |
| *Taurotragus derbianus* | 1000 | Motile | Social | Herbivore | Browser | Vulnerable | Terrestrial | Diurnal | 11 |
| *Sylvicapra grimmia* | 17 | Motile | Solitary | Omnivore | Browser | Least Concern | Terrestrial | Cathemeral | 34 |

Table S2. Home range sizes of recorded mammal species according to Broekman et al. (2022).

| Scientific name | Home range (km^2^) |
| --- | --- |
| *Alcelaphus buselaphus* | 3.25 |
| *Cephalophus rufilatus* | 0.29 |
| *Civettictis civetta* | 5.80 |
| *Colobus guereza* | 0.12 |
| *Crocuta crocuta* | 103.01 |
| *Giraffa camelopardalis* | 282.55 |
| *Hippotragus equinus* | 56.75 |
| *Hystrix* sp. | 0.68 |
| *Chlorocebus tantalus* | 0.53 |
| *Kobus ellipsiprymnus* | 4.00 |
| *Kobus kob* | 0.50 |
| *Lupulella adusta* | 0.85 |
| *Ourebia ourebi* | 0.43 |
| *Panthera leo* | 420.54 |
| *Papio anubis* | 20.90 |
| *Phacochoerus africanus* | 1.96 |
| *Sylvicapra grimmia* | 0.28 |
| *Syncerus caffer* | 114.79 |
| *Taurotragus derbianus* | 66.52 |
| *Tragelaphus scriptus* | 0.17 |
